# Exploring the Therapeutic Potential of Extracellular Vesicles Anchored to the Sea Cucumber Extracellular Matrix for Treating Atopic Dermatitis

**DOI:** 10.34133/bmr.0154

**Published:** 2025-02-21

**Authors:** Sung-Han Jo, Seon-Hwa Kim, Su Chin Heo, Hongsik Cho, Iman Janghorban Esfahani, Sang-Hyug Park

**Affiliations:** ^1^ Department of Industry 4.0 Convergence Bionics Engineering, Pukyong National University, Busan, Republic of Korea.; ^2^ McKay Orthopaedic Research Laboratory, Department of Orthopaedic Surgery, Perelman School of Medicine, University of Pennsylvania, Philadelphia, PA 19104-6081, USA.; ^3^ Department of Orthopaedic Surgery and Biomedical Engineering, University of Memphis, Memphis, TN, USA.; ^4^ Glopex R&D Center, Gyeonggi-do 18469, Republic of Korea.; ^5^ Major of Biomedical Engineering, Division of Smart Healthcare, College of Information Technology and Convergence, Pukyong National University, Busan, Republic of Korea.

## Abstract

Extracellular vesicles (EVs) are crucial for intercellular communication and affect various physiological and pathological processes. Although terrestrial EVs have been extensively studied, marine-derived EVs have yet to be explored. This study investigated the therapeutic potential of sea cucumbers, known for their regenerative and immune abilities. Sea cucumber extracellular matrix (ECM)-anchored EVs (SEVs) were isolated and characterized using physical and electrophoretic analyses. Morphological assessments have shown that SEVs have shape and size distributions similar to mammalian EVs. Internal cargo analysis revealed the encapsulation of diverse proteins and genetic molecules. In anti-inflammatory tests with a lipopolysaccharide (LPS)-induced macrophage model, the results have shown that SEVs can alleviate inflammation factors regarding inducible nitric oxide synthase (iNOS) protein and immune-related mRNA expression. Microarray analysis was conducted to elucidate SEV’s pharmacological efficacy and anti-inflammatory mechanisms, showing that SEVs inhibit the nucleotide-binding oligomerization domain (NOD)-like receptor (NLR) signaling pathway. An in vivo study using a mouse model of atopic dermatitis (AD) induced by 2,4-dinitrochlorobenzene (DNCB) involved subcutaneous SEV administration, followed by severity scoring and histological analyses. Therapeutic efficacy analysis indicated improvements in the AD mouse models, including reduced skin thickness and mast cell numbers. These findings indicate their potential for treating AD. This study highlights the potential clinical applications of marine-derived EVs and offers important implications for future research and therapeutic developments.

## Introduction

Extracellular vesicles (EVs) constitute a diverse group of membrane-bound particles released by cells into the extracellular space, facilitating crucial cell-to-cell communication and molecular exchange. These vesicles actively participate in various physiological and pathological processes within the body [1]. There are 3 types of primary EVs: Exosomes, ranging from 50 to 150 nm in size, originate from the endosomal compartment of cells. EVs are formed when intracellular vesicles carry a specific cargo fused to the plasma membrane, releasing their contents into the extracellular space. Exosomes harbor an array of bioactive molecules, including proteins, lipids, RNA (e.g., microRNAs), and DNA. Microvesicles, larger than exosomes, vary in size from 50 to 500 nm and are generated through outward budding and shedding of the plasma membrane. Analogous to exosomes, microvesicles convey biomolecular cargo and mediate intercellular communication. Apoptotic bodies, measuring approximately 1,000 to 5,000 nm, are released during programmed cell death (apoptosis) and contain cellular fragments and organelles [2]. EVs actively participate in numerous physiological processes, including immune response modulation, tissue regeneration, and development. Consequently, EVs have emerged as promising therapeutic agents for the treatment of arthritis [3], neurodegenerative disorders [4], and cardiovascular diseases [5].

Exosome research predominantly focuses on humans and mammals, accounting for only approximately 6.2% of the entire animal biomass on Earth. Remarkably, marine animals, including arthropods, fish, mollusks, and cnidarians, represent a substantial 77.2% of the animal biomass [6]. However, research on EVs has largely overlooked their roles in marine organisms. This study broadens the scope of the investigation by shifting the focus from terrestrial to marine animals. Biomaterials and substances derived from marine organisms have gained prominence in regenerative medicine and therapeutic applications because of their excellent biocompatibility and unique biological properties [7]. Recent efforts have been made to isolate exosomes from marine organisms [8,9] and explore their biological effects [10]. However, our understanding of the exosomes derived from marine organisms is limited.

Atopic dermatitis (AD) is a prevalent inflammatory skin condition that affects approximately 12% of children and 7.2% of adults worldwide [11]. AD is characterized by hyperproliferation*,* aberrant differentiation of epidermal keratinocytes, and infiltration of immune cell infiltration [12]*.* This study highlights the involvement of NOD-like receptor (NLR) signaling pathways, particularly NOD2 (nucleotide-binding oligomerization domain 2), PYCARD (PYD and CARD domain containing), CARD6 (caspase-recruitment domain 6), IFI16 (interferon-γ inducible protein 16), and NLRP3 (NLR family, pyrin domain–containing-3) inflammasome overexpression in the atopic epidermis [13]. Although AD progression has been extensively studied, current treatment approaches primarily focus on symptom alleviation using topical corticosteroids, immunomodulators, antihistamines, and antibiotics [14]. Extensive research on cell therapy has aimed to improve therapeutic outcomes. Although numerous studies have suggested that mesenchymal stem cell (MSC) therapy can mitigate the progression of AD, it is essential to acknowledge the substantial challenges associated with conducting clinical trials and obtaining Food and Drug Administration (FDA) approval [15]. This is primarily due to concerns regarding the potential for tumorigenesis associated with cell-based therapy, necessitating rigorous safety evaluations. Therefore, exosomal therapy has emerged as an experimental treatment for AD [16]. Exosome therapy offers several advantages, including excellent biocompatibility and therapeutic effects comparable to cell therapy [17]. Furthermore, exosomes can be easily sterilized and have a long shelf life [18], facilitating clinical trials and FDA approval as viable treatment options for AD [19].

**Fig. 1.**
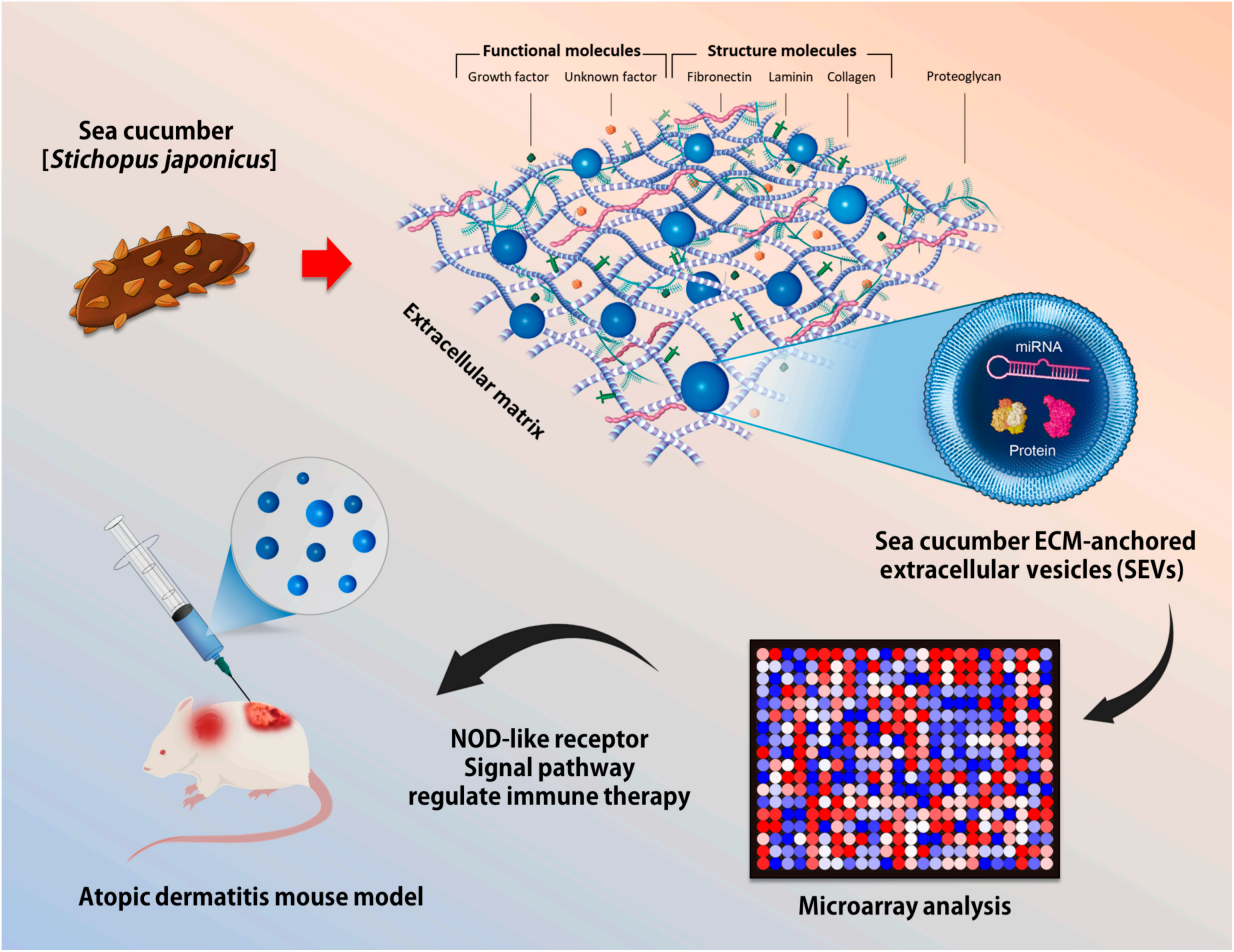
Schematic illustration of sea cucumber extracellular matrix (ECM)-anchored extracellular vesicles (EVs) and their therapeutic effect on atopic dermatitis (AD).

This study focused on the application of marine echinoderm-derived EVs for the treatment of AD. Figure 1 illustrates extracellular vesicles (EVs) anchored to the sea cucumber extracellular matrix (ECM) and their therapeutic effects. Marine echinoderms such as starfish, sea urchins, and sea cucumbers possess unique biological properties, including regeneration ability [20] and immune systems [21,22]. The immune system of sea cucumbers is more complex and robust than that of mammals [23,24]. Several studies have reported the anti-inflammatory effects of sea cucumber-derived biomaterials, including peptides, collagen, and ECM. The peptides have demonstrated strong anti-inflammatory properties, reducing the production of inflammatory cytokines and modulating immune responses. Collagen also plays a significant role in alleviating inflammation and promoting tissue repair. In addition, the ECM derived from sea cucumbers supports cell growth and regeneration while reducing inflammation [25,26]. The authors anticipated that sea cucumber-derived EVs could alleviate this anti-inflammatory environment. Consequently, the optimized extraction process was attempted, but problems were encountered. Although mammalian exosomes are typically isolated from cell culture media or biofluids [27], the use of blood or body fluids from marine organisms is limited because of the pervasive presence of bacteria in seawater [28,29]. Bacteria constitute a significant proportion (~12%) of all life on Earth [6].

When extracting marine-derived EVs, it needs to prevent and exclude contamination with bacteria. Therefore, sea cucumber-derived EVs were isolated from the ECM using a process that minimizes contamination and ensures the isolation of highly pure EVs. We hypothesized that sea cucumbers, which possess a complex immune system, regulate inflammation. ECM-anchored EVs (SEVs) were obtained from sea cucumbers, and their anti-inflammatory effects were evaluated. The mode of action (MoA) and effectiveness were evaluated using large-scale genetic analysis and in vivo applications (Fig. 1).

## Materials and Methods

### Preparation of SEVs

SEVs were isolated as previously described protocols [30,31]. Briefly, the intestine of the sea cucumber (*Stichopus japonicus*) was excised and discarded, and the body of the sea cucumber was frozen. The frozen sea cucumber body was sliced into 1- to 2-cm segments and agitated in a hypotonic buffer at 4 °C for 4 d. The homogenized ECM was centrifuged for 60 min at 8,000*g* and 4 °C. Lyophilized sea cucumber ECM was then incubated with a pepsin solution in HCl (Sigma-Aldrich, St. Louis, MO, USA) at room temperature (RT) for 24 h. The ECM was adjusted to pH 7 with NaOH and dialyzed at 4 °C for 24 h. The resulting ECM suspension was dialyzed and lyophilized. Lyophilized ECMs were treated with collagenase solution (Worthington, OH, USA) in phosphate-buffered saline (PBS) at RT for 2 days to collect SEVs. The enzymatically digested ECM solution was centrifuged to remove the insoluble fraction, followed by filtration through 0.45- and 0.2-μm filters. The filtered solution was purified using ExoQuick-TC precipitation solution (System Biosciences, Palo Alto, CA, USA) according to the manufacturer’s protocol.

### Characterization and cargo analysis

To investigate SEV morphology, physical characterization was performed using transmission electron microscopy (TEM) (H-7500; Hitachi, Tokyo, Japan) and field-emission scanning electron microscopy (FE-SEM) (MIRA 3 LMH; TESCAN, Brno, Czech Republic). The SEV suspension was fixed with 4% paraformaldehyde for 24 h and washed with PBS. Subsequently, SEVs were sequentially dehydrated with 80, 90, 95, and 100% ethanol. The SEV suspension was then dropped onto a cover glass or a carbon-coated grid (TED PELLA, Redding, CA, USA). The SEV sizes were determined using dynamic light scattering (DLS). The SEVs were diluted in 0.2-μm filtered PBS before being placed in an optical-grade cuvette. Size measurements were performed at 25 °C with a size analyzer (Litsizer 500; Anton Paar, Graz, Austria).

The cellular uptake of the SEVs was investigated. The surface membrane was labeled with PKH-26 red fluorescent cell linker (Sigma-Aldrich) for 24 h at 4 °C. Subsequently, the remaining PKH-26 dye was washed 3 times with PBS and then diluted in serum-free RPMI 1640 containing antibiotic–antimycotic to treat RAW 264.7 cells (KCLB, Seoul, Korea) for 12 h. The nucleus was counterstained with Hoechst 33342 fluorescent dye (Invitrogen, Waltham, MA, USA) and washed twice with PBS. A fluorescence microscope (Axio-Observer 5; Carl Zeiss, Oberkochen, Germany) was used to observe endocytosis.

To verify the protein cargo, total SEV proteins were extracted using radioimmunoprecipitation assay (RIPA) lysis buffer (Rockland, Gilbertsville, PA, USA). Protein concentrations were measured using the Bradford assay (Bio-Rad, Hercules, CA, USA). The samples were fractionated on a 4% to 20% sodium dodecyl sulfate (SDS)–polyacrylamide gel (Bio-Rad).

To verify the gene cargo, SEVs were treated with TRIzol reagent (Invitrogen, Grand Island, NY, USA), and the RNA content was measured using a SpectraDrop Micro-Volume Microplate (Molecular Devices, San Jose, CA, USA) at 260 nm. Purified RNA was sent to Macrogen (Seoul, Korea) for analysis using a bioanalyzer (Agilent, Santa Clara, CA, USA) to identify small RNA profiles.

### In vitro anti-inflammatory analysis

Here, we examine the potential anti-inflammatory properties of SEVs. RAW 264.7 cells were seeded in 24-well plates and cultured for 24 h. Subsequently, the cells were treated with SEV suspension (10 μg/ml) in fetal bovine serum (FBS)-free RPMI 1640 medium supplemented with lipopolysaccharide (LPS) (50 ng/ml) (Sigma-Aldrich) for 48 h.

Total RNA was extracted using a spin column extraction kit (Bioneer, Daejeon, Korea) according to the manufacturer’s instructions. Extracted RNA was transcribed into cDNA using a cDNA synthesis kit (CellSafe, Yongin, Korea). Real-time quantitative polymerase chain reaction (qPCR) was conducted using QuantStudio 1 (Thermo Fisher Scientific, MA, USA), employing 2× SYBR Green Reaction Mix (Thermo Fisher Scientific, MA, USA) and specific primers (Table 1). Relative gene expression levels were normalized to those of glyceraldehyde-3-phosphate dehydrogenase (GAPDH), which served as an endogenous control.

**Table 1.** Primer sequences for real-time polymerase chain reaction

Target	Forward sequences (5′-3′)	Reverse sequences (5′-3′)
*TNF-α*	TTCTCATTCCTGCTTGTGGC	GGGAACTTCTCATCCCTTTGG
*IL-1β*	AAAGCTCTCCACCTCAATGG	GCCGTCTTTCATTACACAGG
*IL-6*	GATACCACTCCCAACAGACC	GCAAGTGCATCATCGTTGTTC
*MCP-1*	CCTGCTGCTACTCATTCACC	CTGGACCCATTCCTTCTTGG
*iNOS*	GTGGTGACAAGCACATTTGG	GAACTGAGGGTACATGCTGG
*NF-κB*	AGCCAGCCTTGATGAAGTCT	GATGCCGAGACAAGCTGAAG
*IκBα*	TGAGGGATGACAGGGAAACC	ATAACCTCGCGGAAAACACC
*SOCS-3*	AGGAGAGCGGATTCTACTGG	TGTCGCGGATAAGAAAGGTG
*GAPDH*	TTCAACAGCAACTCCCACTC	TCCTTGGAGGCCATGTAGG

The cells were fixed with 4% (w/v) paraformaldehyde for 15 min at RT and treated with 0.1% Triton X-100 for 10 min. Subsequently, the samples were blocked with 1% bovine serum albumin (BSA) solution for 30 min at RT. After washing, the cells were incubated with an inducible nitric oxide synthase (iNOS)-rhodamine-conjugated antibody, diluted 1:200 in PBS at RT for 1 h. The samples were counterstained with 4′,6-diamidino-2-phenylindole (DAPI). Fluorescent images were captured using a fluorescence microscope (Axio Observer 5; Tokyo, Japan).

### Microarray analysis

Preparation of raw data and statistical analysis: The data were summarized and normalized using the SST-RMA (signal space transformation–robust multi-chip analysis) method implemented in Affymetrix Power Tools (APT). Gene-level SST-RMA and differential gene expression (DEG) analyses were performed. The statistical significance of the expression data was determined using the defined local pooled error (LPE) test and fold change, where the null hypothesis assumed no difference among the groups. The false discovery rate (FDR) was controlled by adjusting the *P* value using the Benjamini–Hochberg algorithm. Hierarchical cluster analysis was performed for each DEG set using complete linkage and Euclidean distance as similarity measures. Gene enrichment and functional annotation analyses of the significant probe list were performed using gene ontology (GO) and the Kyoto Encyclopedia of Genes and Genomes (KEGG). All data analyses and visualizations of differentially expressed genes were conducted using R 3.3.2.

### In vivo analysis

#### Animal model and AD induction

Male BALB/c mice obtained from SAMTACO (Osan, Korea) were used for the AD induction starting at 6 weeks of age. The study was conducted according to animal protocols approved by the Institutional Animal Care and Use Committee of Pukyong National University (PKNUIACUC-2023-05). 2,4-Dinitrochlorobenzene (DNCB) (Sigma-Aldrich) was dissolved in acetone–olive oil (AOO) at a ratio of 4:1, and AOO was used as the vehicle.

#### Induction protocol

The dorsal skin was shaved using an electric clipper and depilatory cream. This was followed by sensitization with 150 μl of 1% DNCB solution on the dorsal skin and ears at −10 and −6 d. Subsequently, a 0.2% DNCB solution was applied at −2 and 0. Mice were challenged with 150 μl of 0.2% DNCB solution thrice weekly for 3 weeks. Concurrently, 200 μl of SEVs (1 and 10 μg/ml) was injected subcutaneously.

#### Evaluation of AD progression

Dermatitis severity was clinically evaluated based on erythema and hemorrhage, edema, excoriation or erosiveness, and dryness and scored from 0 to 3. Total skin severity scores were calculated as the sum of the individual scores (maximum score = 12). Whole blood was collected into a microtube and allowed to clot at RT for 2 h. The samples were centrifuged at 2,000*g* for 10 min to separate serum from the supernatant. All serum samples were frozen immediately at −20 °C until further analysis. The serum immunoglobulin E (IgE) levels were measured using an enzyme-linked immunosorbent assay kit (Thermo Fisher Scientific). The histological evaluation involved fixing back skin samples with 10% formaldehyde, embedding in paraffin, and staining with toluidine blue and hematoxylin and eosin (H&E). The mast cell count and epidermal thickness were analyzed in 5 randomly selected fields using ImageJ software.

### Statistical analysis

GraphPad Prism version 9.3 (GraphPad, San Diego, CA, USA) was utilized to generate graphical representations and conduct statistical analyses. Data are presented as mean ± SD from a minimum of 3 independent experiments. Statistical significance was determined using one-way analysis of variance (ANOVA), followed by a Tukey–Kramer post hoc test. A *P* value of <0.05 was considered statistically significant (*P < 0.05, **P < 0.01, and ***P < 0.001).

## Results

### Characterization of SEVs

**Fig. 2.**
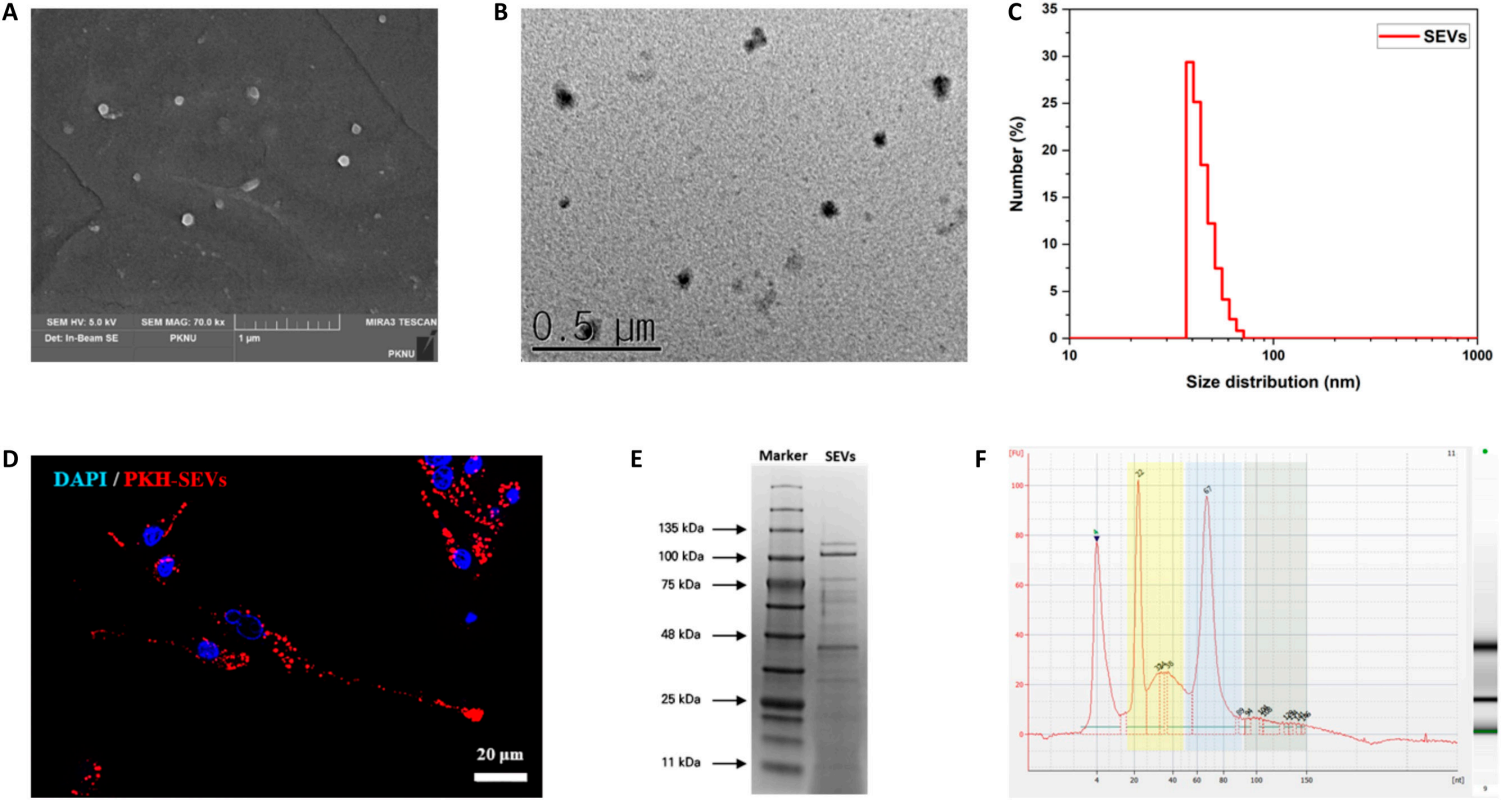
Characterization of SEVs. (A and B) Morphology of SEVs as detected by scanning electron microscopy (SEM) and transmission electron microscopy (TEM). (C) Dynamic light scattering (DLS) was used to profile size distribution. (D) Fluorescence microscopy was used to visualize endocytosis. (E) SDS-PAGE was used to detect protein size distribution. (F) Electropherogram showing the analysis of small RNA content in SEVs. The miRNA and small RNA regions are indicated. The yellow zone (left) represents the miRNA region, the blue zone (middle) represents the transfer RNA (tRNA) region, and the gray zone (right) represents the small ribosomal RNA (rRNA) region.

The electron microscopy images revealed a spherical or round shape (Fig. 2A and B), with a size distribution ranging from 29.38 to 71.42 nm (Fig. 2C). RAW 264.7 cells treated with PKH-26-labeled SEVs displayed successful endocytosis without stimulation (Fig. 2D). The structure and size ranges of these vesicles were consistent with those reported previously for EVs.

### Cargo analysis of SEVs

Proteomic and genomic analyses were conducted to characterize the cargo of SEVs for intercellular communication. SDS–polyacrylamide gel electrophoresis (PAGE) confirmed the presence of diverse protein distributions within the SEVs (Fig. 2E) containing various bioactive substances. Internal genes were identified using a bioanalyzer, which revealed small RNAs of different sizes. The yellow area on the electropherogram indicates the presence of miRNAs with a large amount of 22-nucleotide-long RNA, a common miRNA length, within the SEVs (Fig. 2F).

### Biocompatibility analysis

**Fig. 3.**
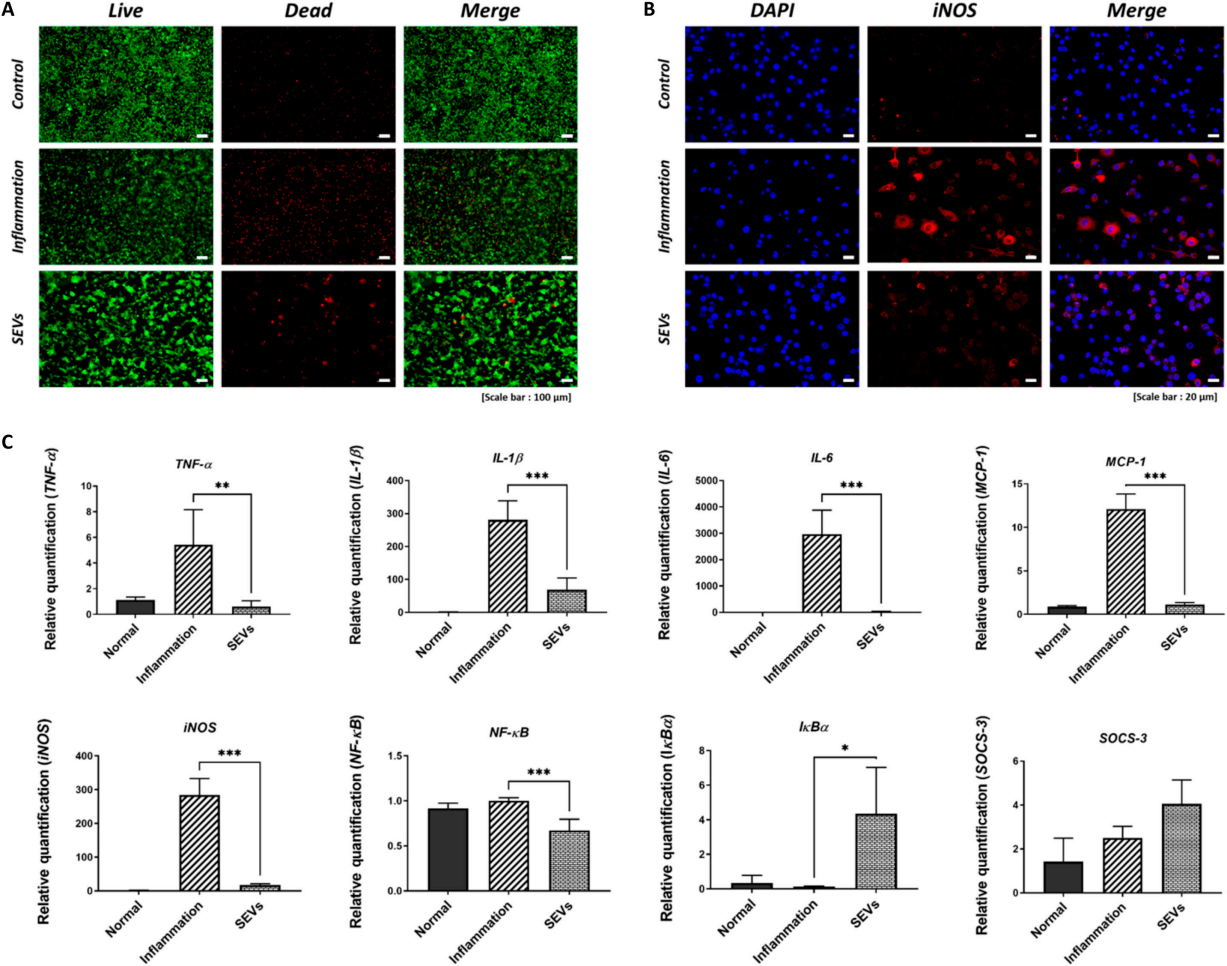
Biocompatibility and anti-inflammatory analysis. (A) Cytotoxicity evaluation with live and dead assay. SEVs (20 μg/ml) were exposed to RAW 264.7 cells for 24 h, with a scale bar of 100 μm. (B) SEVs (10 μg/ml) were exposed to RAW 264.7 cells. After 48 h, immunocytochemistry (ICC) was used to detect iNOS protein expression. Scale bar, 20 μm. (C) Anti-inflammatory activity of SEVs in LPS-induced M1 phenotype model; pro-inflammatory-related mRNA (*TNF-α*, *IL-1β*, *IL-6*, *MCP-1*, *iNOS*, and *NF-κB*) and anti-inflammatory-related mRNA (*IκBα* and *SOCS-3*) expression was analyzed using real-time qPCR and normalized by *GAPDH* endogenous expression. Data are presented as the mean ± SD from independent experiments (*n* = 4) (*P < 0.05, **P < 0.01, and ***P < 0.001, calculated using one-way ANOVA).

The influence of SEVs on cell behavior was assessed by exposing macrophages to LPS (50 ng/ml) and SEVs (10 μg/ml). Compared to the normal and inflammation groups, no significant cell death was observed in the SEV-treated group (Fig. 3A).

### Anti-inflammatory effects of SEVs

Macrophages were treated with SEVs to assess their anti-inflammatory capacity, and subsequent protein and gene expression changes were analyzed. LPS stimulation was used to induce the M1 phenotype in macrophages. After 48 h of SEV treatment, iNOS protein expression was confirmed by ICC analysis, which revealed decreased protein expression in the SEV-treated group (Fig. 3B). Because iNOS serves as a critical downstream mediator of inflammation, its expression is an important indicator of the inflammatory milieu. Furthermore, the expression levels of proinflammatory cytokines and inflammation-related genes were compared. SEVs and LPS were cocultured with RAW 264.7 cells for 48 h, followed by RNA extraction, cDNA synthesis, and real-time PCR. Comparative analysis of pro-inflammatory cytokines and inflammatory factors demonstrated statistically significant suppression in the SEV-treated group compared to the inflammation group. Specifically, tumor necrosis factor-α (TNF-α) was reduced by −89.04% (*P* = 0.005), interleukin-1β (IL-1β) by −75.41% (*P* < 0.001), IL-6 by −99.19% (*P* < 0.001), macrophage chemotactic protein-1 (MCP-1) by −90.05% (*P* < 0.001), iNOS by −93.97% (*P* < 0.001), and nuclear factor κ-light-chain-enhancer of activated B cells (NF-κB) by −33% (*P* < 0.001). Moreover, the comparative analysis of anti-inflammatory factor and cytokine demonstrated significant increment in the SEV-treated group. Specifically, the nuclear factor of κ light polypeptide gene enhancer in B-cells inhibitor α (IκBα) increased by 31.48% (*P* = 0.011) and the suppressor of cytokine signaling-3 (SOCS-3) by 61.7% (*P* = 0.155) (Fig. 3C).

### Microarray analysis

**Fig. 4.**
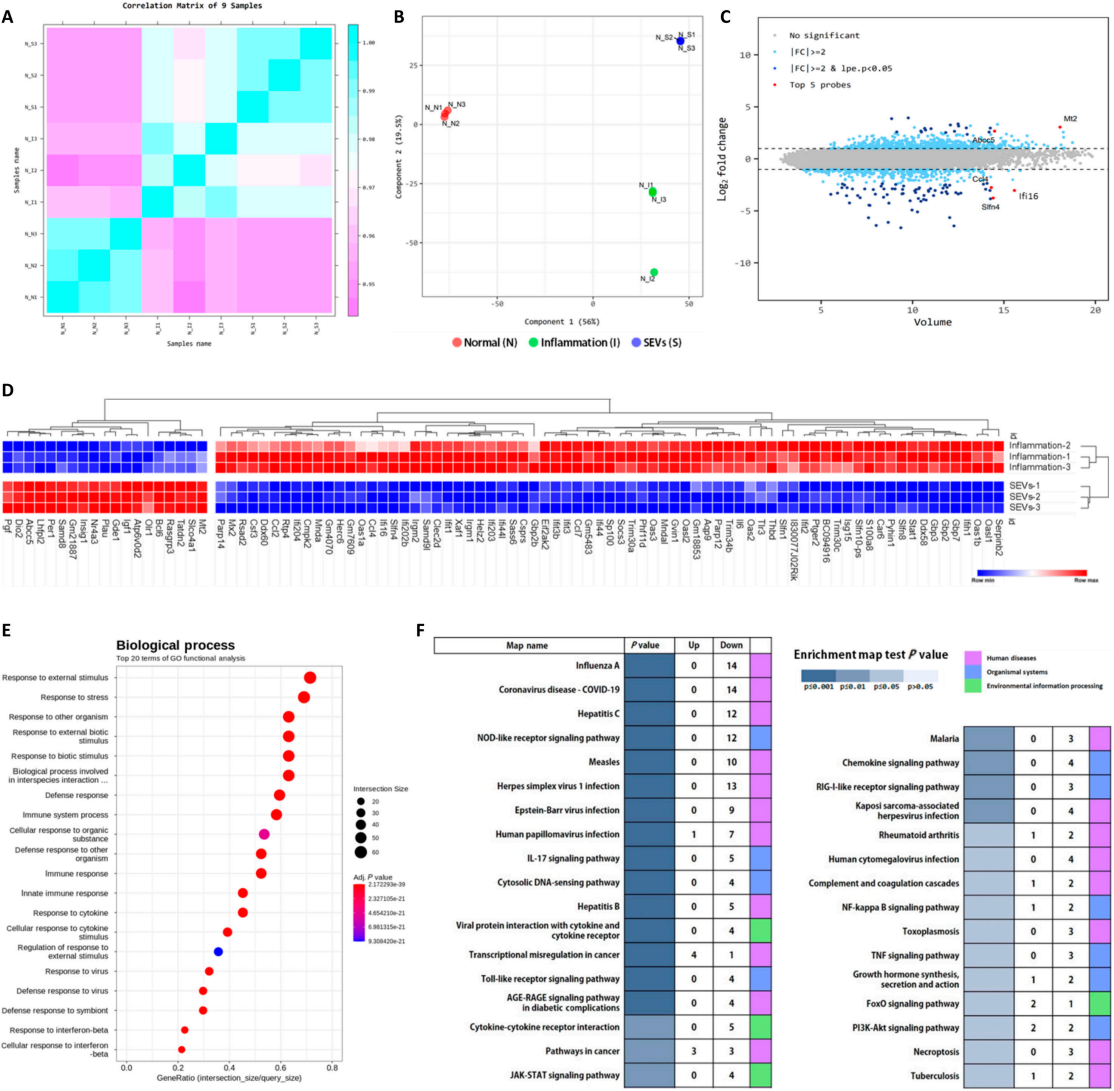
Microarray analysis of the anti-inflammatory effect of SEVs. (A) Pearson’s correlation analysis of samples. The color reflects the intensity of the correlation. The closer the correlation coefficient is to 1, the more it represents blue. (B) Multidimensional scaling (MDS) plot of expression patterns between sample groups. (C) Scatter volume plot of the expression level; the volume (intensity) of the expression value is defined as the geometric mean of the expression values of the 2 groups. The top 5 ranking probes are marked with red dots in order of highest volume. (D) Heatmaps of 92 differentially expressed genes (DEGs) after 48 h of SEV treatment. The figure shows LPS-treated (rows 1, 2, and 3) and LPS/SEV-treated groups (rows 4, 5, and 6) (*n* = 3). (E) Gene ontology (GO) analysis was conducted targeting genes with significant expression level differences. (F) Kyoto Encyclopedia of Genes and Genomes (KEGG) pathway enrichment analysis was conducted using the KEGG Pathway for significant gene expression.

Large-scale genetic analysis was performed using microarray techniques to investigate the MoA of SEVs. Pearson’s correlation test and multidimensional scaling (MDS) were used to assess each group’s overall variation in gene expression patterns (Fig. 4A and B). The Pearson’s correlation analysis results revealed a decrease in correlation values in the SEV-treated group (N_I versus N_S 0.977 ± 0.005, N_I versus N_I 0.990 ± 0.009, and N_S versus N_S 0.994 ± 0.004), indicating alterations in gene expression patterns (Fig. 4A and B). We compared the expression of 22,206 genes between the inflammatory and SEV groups and identified 92 genes with statistically significant differences. Notably, the transcript levels of 73 target genes were down-regulated in response to SEVs, whereas 19 were up-regulated (Fig. 4C and D). Red dots denote the top 5 ranking probes that met the cutoff criteria. Three types of down-regulated genes, C–C motif chemokine ligand 4 (CCL4), schlafen4 (Slfn4), and interferon γ inducible protein 16 (IFI16), and 2 types of up-regulated genes, metallothionein 2 (MT2) and adenosine triphosphate (ATP)-binding cassette subfamily C member 5 (ABCC5), were identified (Fig. 4C).

### GO and KEGG analysis

GO and KEGG pathway enrichment analyses revealed that 727 differentially expressed genes (DEGs) were enriched in 612 GO biological process (BP) terms, 27 GO-cellular component (CC) terms (Table S1), and 88 GO-molecular function (MF) terms (Table S2). These terms included responses to external stimuli, stress, other organisms, biotic stimuli, interspecies interactions, defense responses, immune system processes, cellular responses to organic substances, and responses to viruses (Fig. 4E and Table 2). Furthermore, 308 DEGs were enriched in 33 KEGG pathways, excluding those related to human diseases. These pathways included the NLR signaling pathway, the IL-17 signaling pathway, the cytosolic DNA-sensing pathway, viral protein interaction with cytokines and cytokine receptors, the toll-like receptor signaling pathway, cytokine receptor interaction, the Janus kinase (JAK)–signal transducer and activator of transcription (STAT) signaling pathway, the chemokine signaling pathway, the RIG-I-like receptor signaling pathway, the NF-κB signaling pathway, the TNF signaling pathway, growth hormone synthesis, the FoxO signaling pathway, and the phosphatidylinositol 3-kinase (PI3K)–Akt signaling pathway (Fig. 4F). The analysis highlighted significant associations among inflammatory responses, immunity, and defense responses to other organisms or viruses.

**Table 2.** Top 20 significant gene ontology (GO) terms for biological processes (BP)

Term ID	Term name	*P* value	Up	Down
GO:0051707	Response to other organisms	2.1723E−39	2	51
GO:0043207	Response to external biotic stimulus	2.1723E−39	2	51
GO:0009607	Response to biotic stimulus	4.9802E−39	2	51
GO:0044419	The biological process involved in interspecies interaction between organisms	7.3896E−38	2	51
GO:0009605	Response to external stimulus	1.6696E−35	8	52
GO:0006952	Defense response	2.5303E−35	4	46
GO:0098542	Defense response to another organism	1.2668E−34	0	44
GO:0045087	Innate immune response	2.8484E−31	0	38
GO:0035456	Response to interferon-β	8.0837E−31	0	19
GO:0035458	Cellular response to interferon-β	5.5345E−30	0	18
GO:0034097	Response to cytokines	1.091E−27	0	38
GO:0006950	Response to stress	1.1828E−27	10	48
GO:0009615	Response to viruses	1.5231E−27	0	27
GO:0006955	Immune response	2.172E−27	2	42
GO:0051607	Defense response to viruses	3.6472E−27	0	25
GO:0140546	Defense response to symbionts	3.7246E−27	0	25
GO:0002376	Immune system processes	7.4893E−25	5	44
GO:0071345	Cellular response to cytokine stimulus	4.4729E−23	0	33
GO:0071310	Cellular response to an organic substance	4.7361E−21	5	40
GO:0032101	Regulation of response to external stimulus	9.3084E−21	4	26

### Effects of SEV application in a DNCB-induced AD-like skin mouse model

**Fig. 5.**
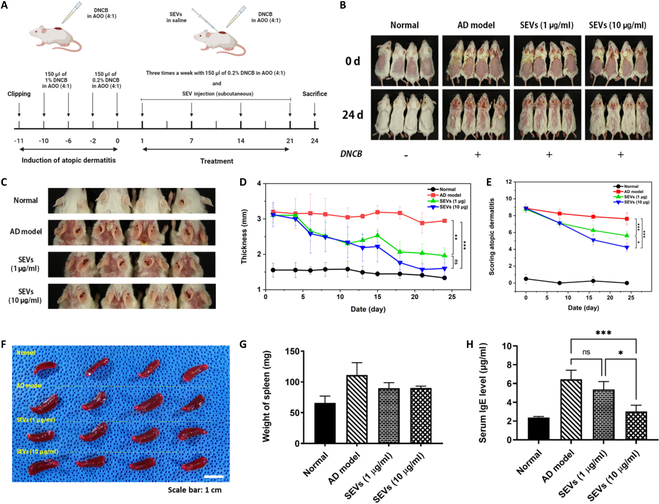
Therapeutic effects of SEVs on AD-like lesions in BALB/c mice model. (A) Study protocol. (B and C) Representative back skin and ear manifestations at days 0 and 24. (D) Skin thickness. (E) Evaluation of clinical dermatitis. The total score is the sum of individual scores determined based on the symptoms of erythema/hemorrhage, edema, excoriation/erosiveness, and dryness. (F and G) Photos and weights of the spleen from the control and SEV groups. (H) Level of IgE. Data are presented as the mean ± SD from independent experiments (*n* = 4) (*P < 0.05, **P < 0.01, and ***P < 0.001, calculated using one-way ANOVA).

The potential ameliorative effects of SEVs on diseases related to the NLR signaling pathway were investigated in vivo. Specifically, the effects of the SEVs were assessed in a murine model of DNCB-induced AD-like lesions. SEVs were administered thrice weekly for 3 weeks (Fig. 5A). We observed dose-dependent alleviation of symptoms in the subcutaneous injection group. The dorsal skin of the AD model mice exhibited significant erythema, edema, excoriation, and dryness. Conversely, the groups treated with SEVs exhibited relief from these symptoms (Fig. 5B). Additionally, ear imaging revealed that SEV application reduced erythema and excoriation (Fig. 5C). Measurement of skin thickness using Vernier calipers indicated a statistically significant decrease in the SEV groups, with higher concentrations showing greater suppression of increased skin thickness [normal: 1.34 ± 0.06 mm, AD model: 2.95 ± 0.04 mm, SEVs (1 μg/ml): 1.96 ± 0.22 mm, and SEVs (10 μg/ml): 1.60 ± 0.27 mm] (Fig. 5D). Clinical dermatitis evaluation scores also exhibited a significant decrease in the SEV group, with a more pronounced decrease observed in the high-concentration SEV group [normal: 0 ± 0, AD model: 7.63 ± 0.75, SEVs (1 μg/ml): 5.63 ± 0.48, and SEVs (10 μg/ml): 4.25 ± 0.50] (Fig. 5E). Compared to the control group, which exhibited worsened symptoms, including erythema, edema, erosion, and dryness of the skin lesions, the SEV treatment group showed improved symptoms. Notably, hypertrophy of immune organs, such as the spleen, indicative of chronic inflammatory skin diseases, was observed. Measurement of spleen size and weight confirmed alterations in immune system status (Fig. 5F and G). Moreover, the AD model showed increased serum IgE levels, whereas the SEV-treated group exhibited a considerable reduction in IgE levels, with approximate reductions of 83 ± 13.1% and 47 ± 10.1%, respectively (Fig. 5H).

### Histological analysis

**Fig. 6.**
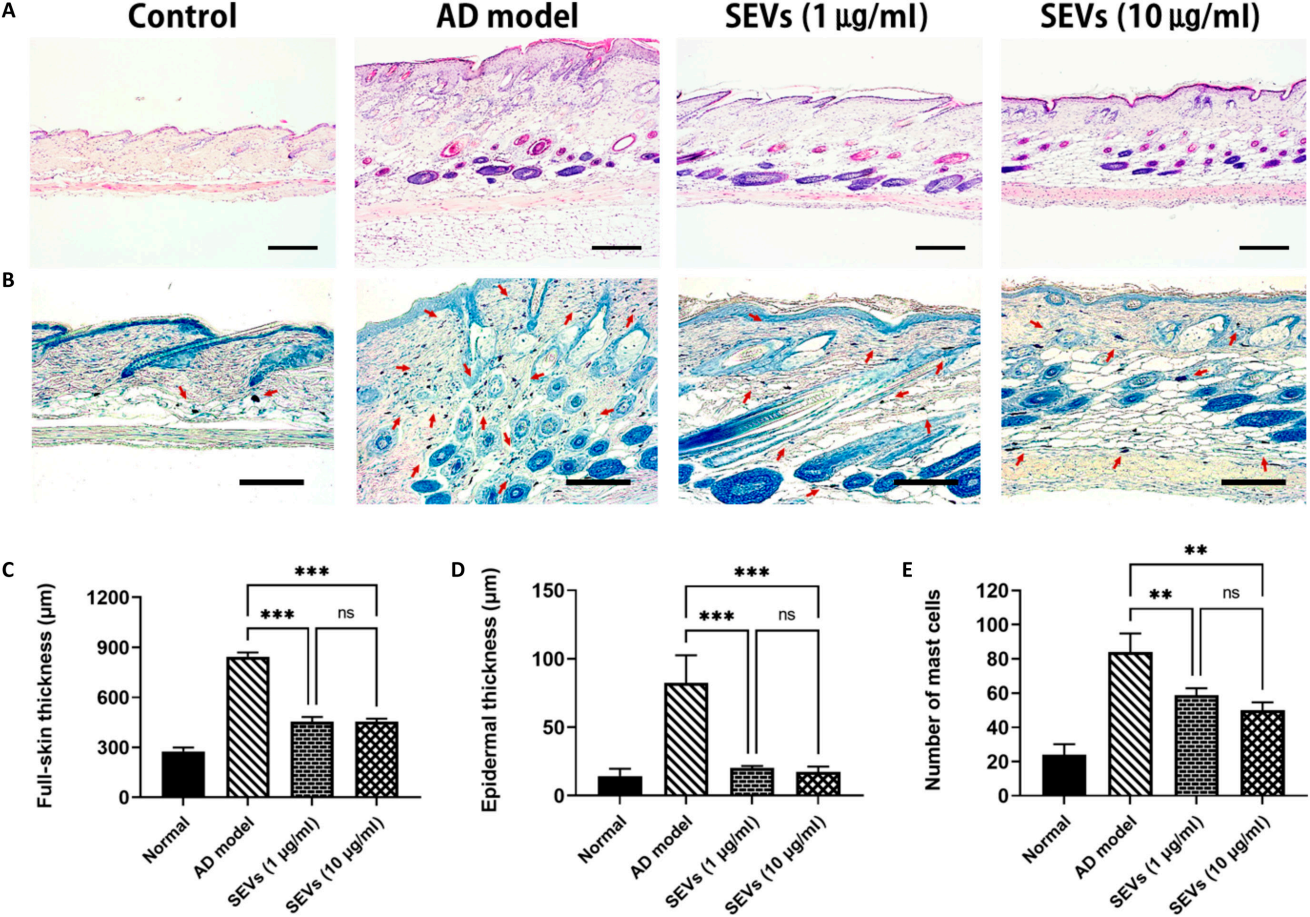
Anti-inflammatory effect of SEVs in histological analysis. (A) H&E staining analysis (scale bar, 200 μm). (B) Toluidine blue staining analysis (scale bar, 100 μm). (C and D) Full-skin and epidermal thickness as measured in H&E-stained tissue sections. (E) Numbers of mast cell infiltration. Data are presented as the mean ± SD from independent experiments (*n* = 4) (**P < 0.01 and ***P < 0.001, calculated using one-way ANOVA).

Histological analysis revealed epidermal thickening and inflammatory cell infiltration (Fig. 6A and B). In the AD model, substantial increases in both overall skin thickness (843.4 ± 25.5 μm) and epidermal thickness (82.4 ± 20.2 μm) were observed. Conversely, the SEV groups demonstrated a significant reduction (*P* < 0.001) in thickness compared with the AD model (Fig. 6A, C, and D). Furthermore, the assessment of inflammatory cell infiltration, particularly mast cell infiltration, demonstrated a dose-dependent effect of SEVs, as evidenced by toluidine blue staining (Fig. 6B and E).

## Discussion and Conclusion

The SEV isolation method was designed to extract EVs from the ECM of sea cucumbers through enzymatic treatment to prevent contamination between EVs or nanoparticles from bacteria and fungi in seawater (Fig. 1). Physical characterization and cargo analysis were performed to determine the presence of exosomes and ECM residue particles (Fig. 2). The observed morphology and size range matched the previously reported values for EVs (Fig. 2A to D). Immunoblot analysis of the surface exosome markers [27] was performed to confirm their identity. Despite efforts to minimize denaturation during the extraction of sea cucumber-derived EVs [30,31], detecting exosome surface markers poses challenges. This issue raised the question of whether this phenomenon could be attributed to potential damage to the surface proteins of SEVs or possible species-specific variations. Subsequent cargo analysis of the SEVs revealed a diverse array of proteins and small RNAs. A wide range of proteins, including large particles such as collagen (110 to 135 kDa) [32], were encapsulated (Fig. 2E). Therefore, we anticipated that SEV proteins and miRNA cargos could be used in regenerative medicine. Various effects of MSC exosomal proteins and microRNAs have also been reported [33,34].

The special biological effects of sea cucumbers are desirable. Sea cucumbers, which contain abundant bioactive compounds, have long been used in traditional medicine in various Asian cultures. Recent research suggests that these bioactive compounds have been extensively studied for their potential use in anti-inflammatory treatments.

In a mouse model of LPS-induced inflammation [35–38], SEVs suppressed the expression of inflammatory cytokines and up-regulated anti-inflammatory factors and cytokines such as IκBα and SOCS-3. These results supported our hypothesis that exosomes expressed by the sea cucumber complex immune system possess anti-inflammatory properties (Fig. 3B and C). A large-scale genetic analysis determined the mechanisms regulating the inflammatory environment. Pearson’s correlation test and MDS results indicated different gene expression patterns (Fig. 4A and B). This pattern change indicated that the SEVs were successfully applied to the inflammation model. Specifically, hierarchical clustering of the enriched terms showed that 73 genes were down-regulated and 19 were up-regulated, with statistically significant differences between the LPS-and LPS/SEV-treated groups (Fig. 4C and D). Among the 92 genes, the top 5 were ranked genes (down-regulated: CCL-4, SLFN4, and IFI16), and the top 5 were up-regulated genes (MT2 and ABCC5).

These 3 down-regulated genes are associated with the immune system. CCL4 attracts specific immune cells to inflammation or infection sites [39], SLFN4 regulates immune responses such as development and function [40], and IFI16 is involved in immune responses [41] and DNA regulation [42]. These 2 up-regulated genes are associated with protection or transport. MT2 is involved in cellular detoxification and protection against oxidative damage [43], and ABCC5 is involved in transporting various molecules across cell membranes [44]. Gene expression was altered to anti-inflammatory and protective states after SEV treatment. In addition, small RNA and protein profiling next-generation sequencing (NGS) and mass spectrometry (MS) were performed to identify the miRNAs and diverse proteins in the SEV.

Despite these attempts, there are limitations due to the absence of a sea cucumber genome library and available MS reference data for echinoderms. This research group is currently conducting experiments to build a gene library and is planning to apply this list of miRNAs to predict diseases in the near future.

GO enrichment analysis revealed that most indicators were related to the ability to defend against external stimuli (Fig. 4E). Remarkable results regarding suppressing the NLR pathway signal were found in 33 KEGG pathway analyses (Fig. 4E and F). The NLR pathway is a critical component of the innate immune system [45] and is the first line of defense against pathogens, such as bacteria, viruses, and other foreign invaders [46]. NLRs are a class of pattern recognition receptors (PRRs) that play crucial roles in pathogen- and damage-associated molecular patterns (PAMPs and DAMPs, respectively) within cells [47]. The NLR pathway is crucial in various autoimmune diseases, including inflammatory bowel disease, rheumatoid arthritis, systemic lupus erythematosus, and AD [12,48]. Among the NLR pathway-related autoimmune diseases, AD involves the overexpression of NOD2, PYCARD, CARD6, IFI16, and the NLRP3 inflammasome. Interestingly, the SEVs suppressed the expression of NLRP3 and IFI16. Therefore, the therapeutic application of SEVs in AD is anticipated to involve suppressing NLR signaling by reducing the expression of NLRP3 and IFI16 genes. Subsequently, an in vivo experiment was conducted to assess the effectiveness of SEVs, which resulted in high therapeutic efficacy. Interestingly, the application of the low concentration of SEVs (1 μg/ml) showed similar therapeutic efficacy compared to high-concentration SEV (10 μg/ml) administration (Figs. 5 and 6). Additionally, AD scoring demonstrated that DNCB-induced AD progression was suppressed by SEVs, leading to improved skin barrier function and reduced pruritus. Similar treatment efficacy was observed at lower concentrations. The IgE expression level related to allergic reactions [49] showed a significant decrease at high concentrations, and there was a gap in immune cell infiltration compared to the inflammation group. Statistically, this effect was observed at high concentrations, and it is encouraging that the therapeutic efficacy was confirmed at low concentrations.

These experiments demonstrated the alleviation effect by focusing only on the NLR signaling pathway. However, autoimmune diseases have complex mechanisms and are not expected to be cured simply by inhibiting the NLR pathway. Therefore, we plan to expand the scope of our research to include relevant signaling pathways in SEV, such as the IL-17 and cytokine–cytokine receptor signaling pathways, to develop more comprehensive treatment approaches.

Our study revealed a significant breakthrough in marine materials, specifically therapeutic EVs derived from the sea cucumber ECM. We successfully optimized a method for extracting SEVs and identified their morphology, which remarkably matched those of mammalian exosomes. Cargo analysis revealed a diverse range of encapsulated proteins and miRNAs.

SEVs were used to treat LPS-induced macrophages, and their biological potential was evaluated. Promising results showed significant suppression of pro-inflammatory cytokine mRNA expression after treatment, accompanied by an increase in the expression of inflammation inhibitors. Moreover, investigation of the underlying mechanism of the anti-inflammatory effects of SEVs revealed statistically significant inhibition of the NLR pathway. In subsequent in vivo experiments, injection of SEVs into the dorsal region of AD-induced mice demonstrated their remarkable effectiveness in alleviating AD progression. Serum and histological staining further confirmed this therapeutic effect, which revealed significant positive outcomes. Our findings provide a promising avenue for treating AD and open up exciting new possibilities for expanding research on EVs derived from marine organisms.

Furthermore, marine organisms from which ECM can be extracted offer new possibilities. The diversity of marine-derived EVs expands our understanding of marine-derived therapeutics. Marine organisms that negatively impact the marine environment, such as jellyfish and starfish, and marine waste, such as fish shells and sea urchin body walls, show potential as a new type of marine resource. In particular, research on SEVs is anticipated to play an important role in marine medicine and as high-value therapeutic agents. This discovery will facilitate groundbreaking advances in marine biology and medicine.

## Data Availability

Please contact the author for data requests.
