# Supplementary material for: Exploring the Therapeutic Potential of Extracellular Vesicles Anchored to the Sea Cucumber Extracellular Matrix for Treating Atopic Dermatitis
Source: Biomater Res. 2025 Feb 21;29:0154. doi: 10.34133/bmr.0154 (PMC11842673; doi:10.34133/bmr.0154)
Supplement: Supplementary 1 — Tables S1 and S2 [file bmr.0154.f1.docx]

**Appendix. Supplementary materials**

**Exploring the therapeutic of extracellular vesicles anchored to the sea cucumber extracellular matrix for treating atopic dermatitis**

Sung-Han Jo^1,3^, Seon-Hwa Kim^1^, Su Chin Heo^3^, Hongsik Cho^4^, Iman Janghorban Esfahani^5^, Sang-Hyug Park^1,2*^

^1^Department of Industry 4.0 Convergence Bionics Engineering, Pukyong National University, 45 Yongso-ro, Nam-Gu, Busan, Republic of Korea

^2^Major of Biomedical Engineering, Division of Smart Healthcare, College of Information Technology and Convergence, Pukyong National University, Busan, Republic of Korea

^3^McKay Orthopaedic Research Laboratory, Department of Orthopaedic Surgery, Perelman School of Medicine, University of Pennsylvania, Philadelphia, PA, 19104-6081, USA

^4^Department of Orthopaedic Surgery and Biomedical Engineering, University of Memphis, TN, USA

^5^Glopex R&D center, B2065, GeumgGangPenterium IX tower, 27, Dongtan cheomdan saneop 1-ro, Hwaseong-si, Gyeonggi-do, 18469, Republic of Korea

^*^ Corresponding author: shpark1@pknu.ac.kr

| **Term ID** | **Term Name** | **p-value** | **Up** | **Down** |
| --- | --- | --- | --- | --- |
| GO:0005737 | cytoplasm | 2.35184E-08 | 10 | 53 |
| GO:0005829 | cytosol | 1.4758E-07 | 4 | 31 |
| GO:0043656 | host intracellular region | 1.41158E-05 | 0 | 3 |
| GO:0033655 | host cell cytoplasm part | 1.41158E-05 | 0 | 3 |
| GO:0033646 | host intracellular part | 1.41158E-05 | 0 | 3 |
| GO:0030430 | host cell cytoplasm | 1.41158E-05 | 0 | 3 |
| GO:0020003 | symbiont-containing vacuole | 1.41158E-05 | 0 | 3 |
| GO:0020005 | symbiont-containing vacuole membrane | 1.41158E-05 | 0 | 3 |
| GO:0033643 | host cell part | 2.19103E-05 | 0 | 3 |
| GO:0043657 | host cell | 0.000183626 | 0 | 3 |
| GO:0018995 | host cellular component | 0.000183626 | 0 | 3 |
| GO:0005622 | intracellular anatomical structure | 0.000594884 | 12 | 57 |
| GO:0110165 | cellular anatomical entity | 0.00074942 | 17 | 63 |
| GO:0005654 | nucleoplasm | 0.001586616 | 3 | 22 |
| GO:0043229 | intracellular organelle | 0.017644027 | 11 | 50 |
| GO:1905370 | serine-type endopeptidase complex | 0.017985316 | 1 | 1 |
| GO:0043231 | intracellular membrane-bounded organelle | 0.018223631 | 11 | 46 |
| GO:1905286 | serine-type peptidase complex | 0.018223631 | 1 | 1 |
| GO:1904090 | peptidase inhibitor complex | 0.024801973 | 1 | 1 |
| GO:0043226 | organelle | 0.026447062 | 11 | 50 |
| GO:0048471 | perinuclear region of cytoplasm | 0.026561868 | 2 | 6 |
| GO:0005576 | extracellular region | 0.030094799 | 4 | 13 |
| GO:0043227 | membrane-bounded organelle | 0.03690953 | 11 | 46 |
| GO:0043230 | extracellular organelle | 0.048401541 | 0 | 3 |
| GO:0033181 | plasma membrane proton-transporting V-type ATPase complex | 0.048401541 | 1 | 0 |
| GO:0065010 | extracellular membrane-bounded organelle | 0.048401541 | 0 | 3 |
| GO:0071682 | endocytic vesicle lumen | 0.048401541 | 0 | 1 |

**Table S1. The significant gene ontology (GO) terms for cellular component (CC).**

**Table S2. The significant gene ontology (GO) terms for molecular function (MF).**

| **Term ID** | **Term Name** | **p-value** | **Up** | **Down** |
| --- | --- | --- | --- | --- |
| GO:0003725 | double-stranded RNA binding | 1.29625E-12 | 0 | 11 |
| GO:0001730 | 2'-5'-oligoadenylate synthetase activity | 1.60715E-10 | 0 | 6 |
| GO:0070566 | adenylyltransferase activity | 2.98928E-07 | 0 | 6 |
| GO:0035639 | purine ribonucleoside triphosphate binding | 0.000136606 | 1 | 19 |
| GO:0017076 | purine nucleotide binding | 0.000152284 | 1 | 19 |
| GO:0097367 | carbohydrate derivative binding | 0.000152284 | 1 | 21 |
| GO:0032555 | purine ribonucleotide binding | 0.000152284 | 1 | 19 |
| GO:0032553 | ribonucleotide binding | 0.000152284 | 1 | 19 |
| GO:0016779 | nucleotidyltransferase activity | 0.000154489 | 0 | 6 |
| GO:0005525 | GTP binding | 0.000170929 | 0 | 9 |
| GO:1901265 | nucleoside phosphate binding | 0.000170929 | 1 | 20 |
| GO:0000166 | nucleotide binding | 0.000170929 | 1 | 20 |
| GO:0005488 | binding | 0.000181566 | 15 | 57 |
| GO:0019001 | guanyl nucleotide binding | 0.000186084 | 0 | 9 |
| GO:0032561 | guanyl ribonucleotide binding | 0.000186084 | 0 | 9 |
| GO:0043168 | anion binding | 0.000197098 | 1 | 21 |
| GO:0097159 | organic cyclic compound binding | 0.000378013 | 6 | 35 |
| GO:0036094 | small molecule binding | 0.000378013 | 1 | 21 |
| GO:0005515 | protein binding | 0.000734161 | 12 | 41 |
| GO:0042802 | identical protein binding | 0.000746512 | 4 | 16 |
| GO:0043167 | ion binding | 0.000844039 | 6 | 30 |
| GO:0005126 | cytokine receptor binding | 0.001244314 | 1 | 6 |
| GO:1901363 | heterocyclic compound binding | 0.001358902 | 4 | 35 |
| GO:0031726 | CCR1 chemokine receptor binding | 0.002174303 | 0 | 2 |
| GO:0031727 | CCR2 chemokine receptor binding | 0.003879623 | 0 | 2 |
| GO:0017111 | nucleoside-triphosphatase activity | 0.004716491 | 0 | 9 |
| GO:0008009 | chemokine activity | 0.005745355 | 0 | 3 |
| GO:0001222 | transcription corepressor binding | 0.006282452 | 2 | 1 |
| GO:0016462 | pyrophosphatase activity | 0.00700355 | 0 | 9 |
| GO:0003924 | GTPase activity | 0.00700355 | 0 | 6 |
| GO:0016817 | hydrolase activity, acting on acid anhydrides | 0.00700355 | 0 | 9 |
| GO:0016818 | hydrolase activity, acting on acid anhydrides, in phosphorus-containing anhydrides | 0.00700355 | 0 | 9 |
| GO:0044389 | ubiquitin-like protein ligase binding | 0.007491276 | 2 | 4 |
| GO:0008270 | zinc ion binding | 0.007491276 | 2 | 7 |
| GO:0001221 | transcription coregulator binding | 0.007683664 | 3 | 1 |
| GO:0048020 | CCR chemokine receptor binding | 0.007683664 | 0 | 3 |
| GO:0003676 | nucleic acid binding | 0.007828067 | 3 | 25 |
| GO:0016772 | transferase activity, transferring phosphorus-containing groups | 0.008234473 | 2 | 8 |
| GO:0019899 | enzyme binding | 0.00874252 | 4 | 13 |
| GO:0005125 | cytokine activity | 0.009858196 | 0 | 5 |
| GO:0008083 | growth factor activity | 0.012368398 | 2 | 2 |
| GO:0003724 | RNA helicase activity | 0.013104243 | 0 | 3 |
| GO:0008186 | ATP-dependent activity, acting on RNA | 0.01386247 | 0 | 3 |
| GO:1990404 | NAD+-protein ADP-ribosyltransferase activity | 0.013985048 | 0 | 2 |
| GO:0048018 | receptor ligand activity | 0.013985048 | 2 | 5 |
| GO:0003677 | DNA binding | 0.013985048 | 3 | 14 |
| GO:0003824 | catalytic activity | 0.013985048 | 5 | 26 |
| GO:0030546 | signaling receptor activator activity | 0.01417248 | 2 | 5 |
| GO:0042379 | chemokine receptor binding | 0.01417248 | 0 | 3 |
| GO:0003950 | NAD+ ADP-ribosyltransferase activity | 0.016972112 | 0 | 2 |
| GO:0016740 | transferase activity | 0.019632071 | 2 | 14 |
| GO:0030545 | signaling receptor regulator activity | 0.020269728 | 2 | 5 |
| GO:0047395 | glycerophosphoinositol glycerophosphodiesterase activity | 0.020269728 | 1 | 0 |
| GO:0005524 | ATP binding | 0.020869135 | 1 | 11 |
| GO:0042803 | protein homodimerization activity | 0.020973834 | 1 | 7 |
| GO:0035035 | histone acetyltransferase binding | 0.020973834 | 1 | 1 |
| GO:0003727 | single-stranded RNA binding | 0.021074949 | 0 | 3 |
| GO:0031625 | ubiquitin protein ligase binding | 0.021074949 | 2 | 3 |
| GO:0003723 | RNA binding | 0.02128198 | 0 | 16 |
| GO:0042887 | amide transmembrane transporter activity | 0.026752853 | 1 | 1 |
| GO:0032559 | adenyl ribonucleotide binding | 0.026752853 | 1 | 11 |
| GO:0030554 | adenyl nucleotide binding | 0.028145628 | 1 | 11 |
| GO:0046872 | metal ion binding | 0.03078621 | 5 | 18 |
| GO:0036431 | dCMP kinase activity | 0.030791953 | 0 | 1 |
| GO:0036430 | CMP kinase activity | 0.030791953 | 0 | 1 |
| GO:0019900 | kinase binding | 0.030791953 | 3 | 5 |
| GO:0033862 | UMP kinase activity | 0.030791953 | 0 | 1 |
| GO:0003690 | double-stranded DNA binding | 0.030791953 | 3 | 9 |
| GO:0005130 | granulocyte colony-stimulating factor receptor binding | 0.030791953 | 0 | 1 |
| GO:0004798 | thymidylate kinase activity | 0.030791953 | 0 | 1 |
| GO:0043169 | cation binding | 0.036647255 | 5 | 18 |
| GO:0015265 | urea channel activity | 0.039658904 | 0 | 1 |
| GO:0005102 | signaling receptor binding | 0.039658904 | 2 | 10 |
| GO:0009041 | uridylate kinase activity | 0.039658904 | 0 | 1 |
| GO:0033798 | thyroxine 5-deiodinase activity | 0.039658904 | 1 | 0 |
| GO:0002950 | ceramide phosphoethanolamine synthase activity | 0.039658904 | 1 | 0 |
| GO:0033188 | sphingomyelin synthase activity | 0.039658904 | 1 | 0 |
| GO:0140657 | ATP-dependent activity | 0.039658904 | 2 | 4 |
| GO:0004800 | thyroxine 5'-deiodinase activity | 0.039658904 | 1 | 0 |
| GO:0046914 | transition metal ion binding | 0.039658904 | 2 | 7 |
| GO:0047493 | ceramide cholinephosphotransferase activity | 0.039658904 | 1 | 0 |
| GO:0001223 | transcription coactivator binding | 0.042007434 | 1 | 1 |
| GO:0016763 | pentosyltransferase activity | 0.048534151 | 0 | 2 |
| GO:0004127 | cytidylate kinase activity | 0.048592885 | 0 | 1 |
| GO:0070851 | growth factor receptor binding | 0.048592885 | 1 | 2 |
| GO:0015254 | glycerol channel activity | 0.048592885 | 0 | 1 |
| GO:0035662 | Toll-like receptor 4 binding | 0.048592885 | 0 | 1 |
| GO:0004694 | eukaryotic translation initiation factor 2alpha kinase activity | 0.048592885 | 0 | 1 |
